# Incorporating otolith-isotope inferred field metabolic rate into conservation strategies

**DOI:** 10.1093/conphys/coae013

**Published:** 2024-04-25

**Authors:** Valesca A de Groot, Clive Trueman, Amanda E Bates

**Affiliations:** Department of Ocean Sciences, Memorial University of Newfoundland, St. John’s, NL, A1C 5S7, Canada; University of Victoria, 3800 Finnerty Rd, Victoria, BCV8 P5C2, Canada; School of Ocean and Earth Science, University of Southampton, Southampton SO1 43ZH, UK; Department of Ocean Sciences, Memorial University of Newfoundland, St. John’s, NL, A1C 5S7, Canada; University of Victoria, 3800 Finnerty Rd, Victoria, BCV8 P5C2, Canada

**Keywords:** ecophysiology, fisheries, in situ, isotope, metabolism, otolith

## Abstract

Fluctuating ocean conditions are rearranging whole networks of marine communities—from individual-level physiological thresholds to ecosystem function. Physiological studies support predictions from individual-level responses (biochemical, cellular, tissue, respiratory potential) based on laboratory experiments. The otolith-isotope method of recovering field metabolic rate has recently filled a gap for the bony fishes, linking otolith stable isotope composition to *in situ* oxygen consumption and experienced temperature estimates. Here, we review the otolith-isotope method focusing on the biochemical and physiological processes that yield estimates of field metabolic rate. We identify a multidisciplinary pathway in the application of this method, providing concrete research goals (field, modeling) aimed at linking individual-level physiological data to higher levels of biological organization. We hope that this review will provide researchers with a transdisciplinary ‘roadmap’, guiding the use of the otolith-isotope method to bridge the gap between individual-level physiology, observational field studies, and modeling efforts, while ensuring that *in situ* data is central in marine policy-making aimed at mitigating climatic and anthropogenic threats.

## Teaser

The otolith-isotope method has the ability to fill the historic field metabolic rate research gap for the bony fishes. The in situ nature of this metabolic metric has transdisciplinary relevance and can provide links to higher levels of biological organisation, informing modelling efforts aimed at mitigating climatic and anthropogenic threats.

## 1. Energy Expenditure and Statement of Purpose

Earth's current geological epoch, the ‘Anthropocene’, is characterized by profound anthropogenic disruption and climate variability (IPCC, 2013)—a threat to biodiversity that is reshaping the natural world at all levels of biological organization. Rapid alterations to natural systems have been examined at the biosphere level, from cascading changes to the hydrological cycle (e.g. Boyer *et al.,* 2007; Lozier *et al.,* 2008), to individual level changes to an organism's energetic budget. Indeed, organismal responses to anthropogenic and climate change occur through physiological (e.g. metabolic rate, osmoregulatory ability, thermal tolerance) and behavioural (predation, competition, symbiosis, parasitism) strategies resulting from altered biological rates at the cellular level (Rilov *et al.,* 2019).

Energy is the currency required for all biological functions, a unifying variable across the animal kingdom. Both the magnitude (food availability) and allocation (physiological costs) of an organism's energy supply to key life sustaining processes therefore influences survival, fitness, species interactions and phenology (Doney *et al.,* 2012; Poloczanska *et al.,* 2013). Measuring how much energy an organism uses provides a mechanistic pathway to predict integrated performance. Metabolic rate is a proxy for the energy expended by an animal over a specific period, and has been studied since the 13th century (Westerhof, 2011). Measuring metabolic rate within the environment can indicate direct impacts of varying environmental factors on metabolism, but also quantify interindividual and interspecific metabolic responses. Differences in metabolic response within a population can provide insight on population viability and food web dynamics, ultimately influencing overall ecosystem structure and function.

The metabolic responses of individuals to complex environmental stressors can highlight important interindividual variability in physiological traits within and across populations, where higher levels of physiological diversity are synonymous with higher resilience, whether that be through resistance or recovery (Spicer and Gaston, 2009). These measurements can provide a reliable real-time assessment of current and future climate change threats on individuals and populations. However, there remains an important disconnect between individual-level physiological response and the multiple environmental stressors associated with global climate change in marine eco-physiological studies. While large-scale multistressor studies have been conducted to examine climate impacts at higher levels of organization, such as species range shifts (e.g. Lauchlan and Nagelkerken, 2020), determining the direct influence of climate driven physiological costs on individual performance is more complex.

To minimize energetic costs associated with environmental change, organisms can adjust their physiology to better perform in a specific habitat—i.e. physiological plasticity (Gaitán-Espitia *et al.,* 2017). Physiological plasticity varies with environmental fluctuations (temperature, pH, salinity, dissolved oxygen, nutrients) with responses in accordance with metabolic genotypes (Harley *et al.,* 2017). Organisms with particular metabolic genotypes are selected for in a population, providing a basis on which natural selection can act (Gunderson and Stillman, 2015; Martínez *et al.,* 2015), and drive interindividual variation in energy budgets based on important energy allocation tradeoffs (Gaitán-Espitia *et al.,* 2017).

**Figure 1:**
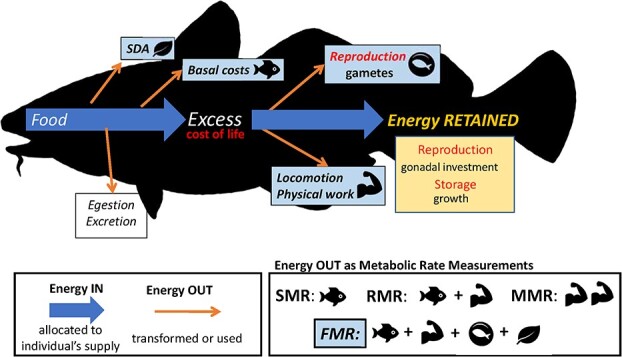
A representation of a fish's energy budget, including Energy OUT, Energy IN, and Energy RETAINED, demonstrating how these energy pathways are included in experimentally measured definitions of metabolic rate (SMR, RMR, MMR, and FMR) using associated symbols. Energy enters the individual as food, and the digestion of this food is associated with the energetic cost of the specific dynamic action (SDA), while energy is further lost through egestion and excretion (nitrogenous waste, carbon and indigestible material not assimilated). The remaining energy is first allocated to basal costs (regulatory functions, body maintenance: SMR), while the excess energy is utilized for reproduction (as Energy OUT and Energy RETAINED), growth (Energy RETAINED), and locomotion/physical work (Energy OUT) for activities such as foraging, predation, and migration (Treberg *et al.,* 2016). Unlike with SMR, RMR, and MMR, FMR (measured using the otolith-isotope method) takes into account one of the first uses of energy as it enters the body—the specific dynamic action (SDA). It also includes basal costs (SMR) and any energy expenditure associated with short-term and long-term activity, such as seasonal movements and reproduction (Activity Metabolism). The ‘double muscle’ symbol for MMR refers to purposefully enhanced activity. (Atlantic cod silhouette: Cerren Richards).

Energy budget modelling can provide a mechanistic understanding of environmental change. For example, a heterotrophic organism's energy supply is synthesized from organic food molecules (Hill *et al.,* 2012) and is then allocated to key life processes as: supply (Energy IN), transformation or use (Energy OUT) and growth or reproductive effort (Energy RETAINED; Fig. 1). Energy is transformed from organic food molecules through the process of glycolysis, producing the organic compound adenosine triphosphate (ATP) which provides energy to drive crucial body processes, such as chemical synthesis and muscle contraction (Hill *et al.,* 2012; Nelson, 2016). The ultimate indicator of energy expenditure is the heat generated by the body measured using direct calorimetry, however, this method has several disadvantages, including the complex engineering, costs and appropriate facilities required to build whole-room calorimeters (Johnson and Coward-McKenzie, 2001). In lieu, indirect calorimetry has been widely adopted by physiologists, kinesiologists and ecologists alike, using oxygen consumption (and carbon dioxide production) as a measurable indicator of ATP energy. Evolutionary, ecological and physiological aspects of metabolic rate can be examined at various levels of biological organization including: 1) individuals (tissue and cellular respiration rates, reproduction, growth, performance, mortality); 2) populations (productivity, predator–prey interactions, population dynamics, differential energy expenditure patterns); and 3) ecosystems (resource depletion, trophic dynamics) (Brown *et al.,* 2004; Chabot *et al.,* 2016).

**Table 1:** Laboratory measurements of different metabolic rate metrics (SMR, RMR, MMR, FMR, AS) for fishes, their biological meaning, the required condition of the organism at measurement, and the methods of measurement commonly used (Blažka *et al.,* 1960; Brett, 1964; Beamish, 1978; Steffensen *et al.,* 1984; Killen *et al.,* 2010; Killen *et al.,* 2012; Metcalfe *et al.,* 2016; Treberg *et al.,* 2016; Killen *et al.,* 2016b; Killen *et al.,* 2016a; Norin and Clark, 2016; Nelson, 2016).

**Metabolic Measurement**	**Biological Meaning**	**Conditions for Measurement**	**Methods Examples**	Fig. 1 **Pathway**
Standard Metabolic Rate (SMR)	Baseline metabolic rate needed to sustain life in an ectotherm by maintaining organismal homeostasis (protein and DNA repair, maintenance of ion gradients, ATP production, anabolism and catabolism of tissues, etc.)	**Rest** (coaxed to truly minimal levels of activity) **Post-absorptive state** (SDA effects absent) Knowledge of the ambient **temperature** at which measurements occurred*	Continuous Flow Respirometry: oxygen consumption measurement	Basal costs
Routine Metabolic Rate (RMR)**	Baseline costs (SMR) + normal spontaneous activity, maintenance of posture/equilibrium or voluntary activity. Behaviour/activity must be carefully specified and defined, and the amount of activity should be quantifiable	Influenced by **random activity** under experimental conditions **Post absorptive state** (SDA effects absent) **Protected from outside stimuli**	Continuous flow respirometry: oxygen consumption measurement	Basal costs + Any specified random activity
Maximum Metabolic Rate (MMR)	The upper threshold of metabolic capacity attainable by the individual at a given temperature under any ecologically relevant circumstance	Fish exposed to **critical swimming speed tests**, **burst-swimming protocols**, and **exhaustive chases** until a defined critical swimming speed has been reached (point of fatigue, max O_2_ consumption) **Post absorptive state** (SDA effects absent)	Blažka-, Brett- and Steffensen-type swim flume respirometers	Enhanced activity (locomotion/work)
**Field Metabolic Rate (FMR)** ***	Baseline costs (SMR) + specific dynamic action (SDA) + and activity metabolism. Represents time-averaged energy intake and expenditure	Measured on a free-ranging organism in its natural habitat, undisturbed by confinement and manipulation	Otolith-isotope method	Basal costs (SMR) + SDA + Activity (locomotion/work)
Aerobic Scope (AS)	Difference between MMR and SMR under the same environmental conditions. Defines the overall capacity of the animal to increase its metabolism in relation to non-regulatory requirements, such as locomotion for migration, energy for predation, reproduction, etc	N/A	Measured MMR - SMR under the same laboratory conditions **OR** FMR - SMR under the same field conditions	Enhanced activity (locomotion/ work) - Basal costs **OR** Field Metabolic Rate - Basal costs

The energy pathway each measurement represents can be followed in Fig. 1.

^*^As opposed to the thermoneutral zone assumption in Basal Metabolic Rate (BMR) measurements for endotherms.

^**^Additional caution should be applied when using the term RMR for fishes, as its definitions can be somewhat vague and can sometimes be used interchangeably with SMR.

^***^Energy pathways demonstrated for field metabolic rate measured using the otolith-isotope method.

The response of marine fishes to changing temperature has received much research attention due to their physiological vulnerability as ectotherms (e.g. Rose *et al.,* 1994). Fluctuating temperatures destabilize the biological processing rates of fishes, which can cause cascading shifts to organisms (energy budgets), populations (productivity), communities (species-interactions) and ecosystems (food web structure). To date, physiological processes of fishes have been advanced through extrapolation from observations of historic populations or from laboratory-based measures of respiratory potential (Table 1). For instance, laboratory multistressor studies replicate current and projected environmental factors (Schwieterman *et al.,* 2019) and measure factors such as aerobic scope alteration and hypoxia tolerance as indicators of responses to environmental stress. While multistressor studies can highlight the interactive effects of varying environmental stressors, several crucial spatiotemporal factors (e.g. seasonality, life history, phenology, local adaptation) and co-occurring drivers (nutritional status, thermal tolerance, oxygen availability, food availability, competition, predation) associated with complex natural habitats might enhance or buffer responses between individuals in an ecosystem (e.g. Crain *et al.,* 2008; Kuo and Sanford, 2009; Doney *et al.,* 2012; Graiff *et al.,* 2015; Crisci *et al.,* 2017; Rilov *et al.,* 2019). Even after controlling for sources of variation related to lifestyle (e.g. benthic vs pelagic), metabolic rate can differ by a factor of up to three among individual fish of the same species, sex and age held in similar conditions (Millidine *et al.,* 2009; Norin and Malte, 2011; Killen *et al.,* 2012).

**Table 2:** Summary of historical FMR methods (Lifson *et al.,* 1949; Lifson *et al.,* 1955; Priede and Young, 1977; Burger, 1981; Karasov, 1983; Shoemaker and Nagy, 1984; Nagy and Shoemaker, 1984; Gales *et al.,* 1988; Nagy, 1989; Lucas *et al.,* 1991; Nolet *et al.,* 1992; Brekke and Gabrielsen, 1994; Boyd *et al.,* 1995; Berteaux *et al.,* 1996; Thorarensen *et al.,* 1996; Wolf *et al.,* 1996; Nagy *et al.,* 1999; Speakman 2000; Gabrielsen and Ellis, 2001; Nagy, 2001; Ward *et al.,* 2001; Butler *et al.,* 2004; Nagy, 2005; Snelderwaard *et al.,* 2006; Wilson *et al.,* 2006; Acquarone *et al.,* 2007; Plasqui and Westerterp, 2007; Whitney *et al.,* 2007; Sparling *et al.,* 2008; Green *et al.,* 2009; Clarke *et al.,* 2010; Gräns *et al.,* 2010; Enstipp *et al.,* 2011; Green, 2011; Halsey *et al.,* 2011; Hudson *et al.,* 2013; Elliott *et al.,* 2013; Dalton *et al.,* 2014; Scantlebury *et al.,* 2014; Wright *et al.,* 2014; Stothart *et al.,* 2016; Treberg *et al.,* 2016; Rojano-Donãte *et al.,* 2018; Pagano and Williams, 2019)

*Method*	**Time energy budget**	**Accelerometry**	**Heart Rate Telemetry**	**Doubly Labeled Water**
*Period of measured FMR*	Timescale of observation	Total time of transmitter tracking (days to months)	Total time of transmitter tracking (days to months)	Time of species specific isotope washout rate (24 h—28 days)
*FMR temporal resolution*	Observation error associated with changes in activity limit fine scale FMR information	Fine temporal scales	Fine temporal scales (minute to minute changes in FMR)	Only available over a large temporal scale, (FMR average per 24 h period)
*Applicable to*	Terrestrial animals, continually observed	Terrestrial and aquatic animals, limited by tag size	Terrestrial and aquatic animals, limited by tag size and surgical vulnerability	Terrestrial vertebrates and invertebrates, aquatic mammals, no size limit
*Pros*	Minimal field equipment required Low cost	Combine with environmental loggers to track abiotic factor influence on energy output Measures variation in short-term activity and behaviours	Combine with environmental loggers to track abiotic factor influence on energy output	FMR value encompasses all metabolic pathways Insight at various levels of biological organization (organ systems to daily food requirements and trophic dynamics)
*Cons*	**No direct metabolic rate measurements:** Uncertainty in basal metabolism to activity metabolism conversions Limited to tracking **short-term** energy trends **Organisms must remain in sight**: bias towards organisms/life cycles that are tied to specific sites (nesting/breeding) **High sampling effort/high probability of observer error**: continual observation and good knowledge of activity/behavior required	**Uncertainty in O**_**2**_ **consumption conversion**: Variation in muscular efficiency between species Limited to tracking **short-term** energy trends **Changes in behavior related to surgical stress and externally attached devices**	**Uncertainty in O**_**2**_ **consumption conversion**: Inconsistent relationship between heart rate and cardiac output across metabolic stimuli Limited to tracking **short-term** energy trends **Changes in behavior related to surgical stress**	**Additional uncertainty associated with CO**_**2**_ production conversion*: Unknown species-specific respiratory quotient increases variability in conversion values compared to O_2_ consumption conversions. Limited to tracking **short-term** energy trends **Potential changes in behaviour associated with method handling** **Washout rate imposes a narrow recapture window:** potential need for back calculations, increased cost of study, limited sample sizes

It is crucial to measure an individual's metabolic rate under the influence of real abiotic and biotic factors present in its environment to both capture the direct impacts of varying environmental factors on metabolism, while also targeting interindividual and interspecific metabolic responses that might interact to reorganize communities in unpredictable ways (e.g. Dong *et al.,* 2017). Experimental systems also typically expose individuals to conditions outside of those in the population's natural experience, therefore, it is currently unclear whether acclimation over laboratory timescales is sufficient to replicate adaptive buffering potential of multi-generational exposure to a given condition. Metabolic enzyme activity and RNA/DNA have been used as biochemical indicators of metabolic condition in free-ranging marine invertebrates and fish, representing direct change in environmental condition (Dahlhoff, 2004; Lesser, 2016). While this method represents an important *in situ* measurement of metabolic rate, it yields a snapshot of the condition of the individual at the time of sampling, with no accompanying spatiotemporal history of metabolic experience or environmental exposure (Dahlhoff, 2004). This is a crucial gap, as realistic predictions on the fate of species and populations require accurate indicators of experienced environmental conditions. This has become especially apparent in recent studies, highlighting that the frequency, intensity, and sequence of abiotic change might be more important to the alteration of population dynamics and species distribution (Pearson *et al.,* 2009) than changes in mean conditions (Wahl *et al.,* 2013; Benedetti-Cecchi *et al.,* 2015). To strengthen climate predictions, scientists require a tool that provides time-integrated *in situ* data on both the individual's physiological response (metabolism) and the associated spatiotemporal indicators of experienced abiotic factors (Bates *et al.,* 2018; Kelly, 2019).

Here we describe a novel way of acquiring real-time metabolic rate data for free-ranging fishes (field metabolic rate, FMR) through the isotope analysis of sagittal otoliths (Chung *et al.,* 2019b). We highlight the unique opportunity provided by the otolith record, which combines quantitative data on individual variation in metabolic traits with a time-integrated spatiotemporal record of experienced water chemistry, creating a metabolic indicator of climate and anthropogenic change. The purpose of this paper is three-fold: 1) to compare standard measures of metabolism in fish to field metabolic rate opportunities historically confined to terrestrial organisms; 2) to highlight the otolith-isotope method as a novel and robust tool to acquire individual, time-integrated measures of field metabolic rate for fish; and 3) to establish a new definition of metabolism in the multidisciplinary fields of fish biology, encouraging a new data standard for the creation of powerful predictive models. Understanding the physiological mechanisms that are modifying and reshaping communities will improve biological forecasting on climate change threats to ecosystems and the services they provide. The ability to integrate *in situ* data on the physiological plasticity of free-ranging fish into models at various levels of biological organization has the potential to better inform conservation objectives and management strategies under current and projected climate scenarios.

## 2. Techniques for Measuring Metabolic Rate

An organism's realised field metabolic rate (FMR) describes the energy expenditure of a free-ranging organism in its natural environment, averaged over the duration of the observation period. Assessing FMR in free-living organisms avoids confounding factors associated with laboratory studies, such as the complications associated with acclimation and the stresses of manipulation. Furthermore, it provides an opportunity to assemble the context-dependent measures of metabolism associated with life in specific habitats, including the energetic trade-offs associated with complex ecological and environmental interactions. FMR may be a more ecologically relevant measure of energy expenditure, but as a composite measure of basal and active energetic processes, variations in FMR may be complicated to interpret. FMR encompasses different energetic pathways based on the method of measurement used. Here, we focus on the **otolith-isotope method**, while alternatives will be explored in the following section of this review.

The measurement of FMR encompasses the regulatory maintenance functions associated with standard metabolic rate (SMR) and daily activities ranging from foraging to locomotion (Smith, 1980), while also including one of the first uses of energy, the specific dynamic action (SDA) (Fig. 1; Table 1). The SMR represents the baseline metabolic requirements for maintaining organismal homeostasis, while activity metabolism measures the energy allocated to foraging, feeding, predation, locomotion, and behaviours associated with reproduction, such as courtship and parental care (guarding, scattering). The specific dynamic action (SDA) is related to digestion and is defined as the increase in oxygen consumption following a meal. Summing these three metabolic metrics, field metabolic rate represents a time-averaged or instantaneous measure of energy intake and expenditure. While this measure represents an integrated energetic measure of the organism's response to its environment, depending on the research question, the holistic nature of the FMR value could present a drawback due to the difficulty in separating basal from active energetic contributions (SMR, SDA, activity).

The inclusion of both maintenance metabolism (SMR) and activity metabolism in the measure of field metabolic rate highlights the critical physiological tradeoffs that prioritize survival in the face of fluctuating ocean conditions (e.g. water temperature, acidification, hypoxia events) and how critical thresholds may in turn alter activity through physiological and behavioural plasticity (Treberg *et al.,* 2016; Chung *et al.,* 2021). Activity metabolism varies dramatically between species based on the organism's lifestyle characteristics, influencing both the size of the metabolic scope as well as the baseline SMR associated with the metabolic cost of maintaining high-activity machinery (Priede, 1985; Norin and Malte, 2012). While the threshold of activity for a species can be determined in a laboratory setting (MMR), it is the daily activity of an individual interacting with its direct environment and how survival mechanisms controlled by SMR can prioritize these interactions, which makes activity metabolism interesting within the scope of FMR.

Specific dynamic action (SDA) is a key parameter to factor into the interpretation of FMR, as it is a cost that is not included in SMR or activity metabolism, yet, is dependent on the environment in which the individual lives. The SDA of an organism is driven by the biochemical processes involved with the digestion and mobilization of amino acids (Kleiber, 1961). SDA can vary between 5–20% depending on the organism's diet, with high-protein diets producing the highest SDA values (Priede, 1985). Food quality and quantity can further alter the energy costs associated with SDA. For instance, the seasonality of food resources can lead to variations in the allocation of energy towards the various metabolic pathways contained within the estimated FMR value, from SDA, to somatic growth, activity and reproduction. Fish that live in environments with excess food resources are known to have an increased metabolic cost of digestion, creating a metabolic conflict in energy budgeting by limiting performance (e.g. inhibited swimming activity) and prioritizing digestion (Priede, 1985). The incorporation of SDA within the measurement of FMR in free-ranging fishes therefore holds a two-fold benefit as it provides integrated information on (1) resource distribution and composition in an organism's environment (high SDA—abundant resources/high protein diet, low SDA—limited resources/low protein diet) and, (2) the reallocation of energy associated with SMR and activity metabolism (high SDA—activity limiting, low SDA—activity priority).

The measurement of field metabolic rate differs from metabolic rate methods that have thus far dominated the field of fish experimental physiology and have subsequently informed the fields of ecology and behaviour (Table 1). Studying SMR, MMR, and aerobic scope allows us to gain mechanistic understanding of ecophysiological responses to specific drivers. While these measures of metabolism represent fundamental components of physiology functioning, they are unlikely to reflect realized physiological responses to complex environmental stressors. These metabolic metrics also contain inherent limitations in their application to free-ranging fishes. Measurements of SMR, for example, are only taken at a single point in time so that variation between individuals can represent measurement errors and random temporal fluctuations, therefore, true differences in regulatory energy expenditure are not always clear (Metcalfe *et al.,* 2016). Methods of MMR measurements also differ between species of fish, for instance, which vary in their willingness to swim against water currents, making it difficult to exercise them to exhaustion (Norin and Clark, 2016).

### 2.1 How Has Field Metabolic Rate Historically Been Measured?

Despite the prior definition of FMR, the term ‘field metabolic rate’ can have different meanings across studies based on the method each uses. Thus, different studies might yield slightly different information for the same species, as they account for different energetic pathways of metabolizable energy in free-ranging animals (Fig. 1). Historic methods of field metabolic rate measurements include time energy budgets, accelerometry, heart rate telemetry and doubly labeled water (Table 2). Doubly labeled water has been termed the ‘gold standard’ for the determination of field metabolic rate and can be conducted on air-breathing organisms with a large range in body size, e.g. from walruses to bumblebees, a difference in size of a magnitude of 10^6^ (1310 kg to 0.0003 kg) (Wolf *et al.,* 1996; Butler *et al.,* 2004; Acquarone *et al.,* 2007; Plasqui and Westerterp, 2007).

#### 2.1.1 Doubly Labeled Water: Inapplicable to the Largest Class of Vertebrates—The Bony Fishes

The primary concern with the doubly labeled water method is that it has not been transferrable to aquatic species due to its high whole body water turnover rate (Motais *et al.,* 1969; Maloiy, 1979). In water-breathing and amphibious organisms, labelled oxygen in body water is quickly removed, while very little isotopic oxygen leaves the animal as CO_2_ (Nagy, 1989). For the DLW method to work, a substantial fraction (~15%) of isotopic oxygen needs to leave the animal as CO_2_. In teleost fish, however, unidirectional water influx rates cause body water turnover rates as fast as 10% to 100+% per hour and osmoconforming species, such as salmonids and anguilliformes, can even exceed this (Nagy, 1989; Schmidt-Nielsen, 1997). Because of the smaller ratio of CO_2_ production to water, the error involved in detecting and quantifying CO_2_ production in water-breathing and amphibious animals increases dramatically.

Acquiring metabolic information on fish, both bony (teleost, lungfish) and cartilaginous (sharks, skates and rays), has therefore been restricted to methods, such as electromyogram telemetry (Rogers and Weatherley, 1983; Hinch *et al.,* 1996; Briggs and Post, 1997; Cooke *et al.,* 2004; Quintella *et al.,* 2009), heart rate monitoring (Priede and Young, 1977; Priede, 1983; Priede, 1985; Lucas *et al.,* 1991; Sureau and Lagardére, 1991; Thorarensen *et al.,* 1996; Clark *et al.,* 2005), ventilation frequency (Frisk *et al.,* 2012), tail beat frequency (Ross *et al.,* 1981), acoustic tri-axial accelerometer transmitters (Kaseloo *et al.,* 1992; Metcalfe *et al.,* 2016), intermittent-flow respirometry (Murchie *et al.,* 2011), field-based respirometry (Bailey *et al.,* 2002; Farrell *et al.,* 2003) or a combination of these (Barnett *et al.,* 2016). These approaches only yield short-term ‘snapshots’ into the energy budget of free-ranging fish (Martino *et al.,* 2020). The review by Treberg *et al.* (2016) summarizes an array of available methods and their potential to capture the routine energy expenditure of bony fishes in the field. An important advancement has been made since this publication regarding the quantifiable link between biomineral carbon in fish otoliths and oxygen consumption (Chung *et al.,* 2019b).

## 3. The Otolith-Isotope Method and Field Metabolic Rate

### 3.1. **The Otolith: The Fish**'**s Spatiotemporal Record**

The otolith is an acellular, unvascularised, incrementally grown structure. Its acellular nature means that once deposited, otolith mineral cannot be resorbed. The otolith therefore provides a lifetime record of the chemical environment in the endolymph fluid at the time of deposition (Reis-Santos *et al.,* 2023). Three bilaterally symmetric sets of otoliths (the sagittae, lapilli and asterisci) are located in the endolymph fluid of the vestibular apparatus of the fish's inner ear and are involved in sensory perception (Solomon *et al.,* 2006). In many fishes, the asteriscus may be made of vaterite, and pathological vaterite deposition is commonly reported in some taxa such as salmonids. Comparative chemical approaches assume that aragonite mineral is sampled, therefore, if the presence of vaterite is suspected, otoliths should be examined carefully and avoided if confirmed (Tomas and Geffen, 2003). The precipitation rate of aragonite (crystal CaCO_3_) from the endolymph fluid is linked to fish metabolism via production of CO_2_ and bicarbonate. Increased bicarbonate concentrations in blood facilitate export of bicarbonate into the endolymph and therefore increases mineral growth. Consequently, otolith growth is linked to daily, seasonal, and lifelong variations in metabolism and growth. These variations in mineral growth rates are expressed in the otolith through variations in the relative concentrations of mineral and organic protein-sugar matrix components, leading to the otolith bands widely used in fish ageing (Campana, 1999). In a crosswise section, the otolith appears to have opaque and translucent bands due to variations in the proportion of mineral compared to organic constituents (organic-rich layers absorb more light. These alternating bands of organic-rich and organic-poor layers are deposited in accordance with daily and annual growth increments providing morphological information on age and growth, while the isotopic composition of the associated mineral yields information on experienced water chemistry (δ^18^O value) as well as diet and metabolic expenditure (δ^13^C value). The isotopic composition of both oxygen and carbon in otolith aragonite have historically been used to quantitatively link a fishs' individual biochemistry to its direct environment, providing insight into dietary composition (Nonogaki *et al.,* 2007; Elsdon *et al.,* 2010; von Biela *et al.,* 2015), population structure (Ashford and Jones, 2007), geography, migration and spatial ecology (Secor *et al.*, 1995; Ashford and Jones, 2007; Trueman *et al.,* 2012; Kimirei *et al.,* 2013; Javor and Dorval, 2014; Currey *et al.,* 2014; Gerard *et al.,* 2015), as well as stock identification and life history ecology (Gao and Beamish, 1999; Gao *et al.,* 2001; Bastow *et al.,* 2002; Gao *et al.,* 2010; Correia *et al.,* 2011; Shen and Gao, 2012). Stable isotopes of oxygen undergo equilibrium fractionation during incorporation into precipitating aragonite, with the fractionation effect predictably related to the temperature of precipitation (Kim and O'Neil). The thermal sensitivity of equilibrium fractionation of oxygen isotopes between water (endolymph) and aragonite appears to be conserved across fish taxa and is not measurably different from that of inorganic aragonite precipitation (Morissette *et al.,* 2023). However, the isotopic offset between ambient water and otolith aragonite; the intercept term in isotope thermometry equations, does appear to vary among taxa and environment (freshwater vs marine), potentially related to differences in physiology and/or water balance. A meta-analysis of biomineral oxygen isotope fractionation relationships (Morissette *et al.,* 2023) suggests the following two otolith thermometry equations if species-specific intercept terms are unavailable:

Freshwater: δ ^18^O_otolith,VPDB_ - δ^18^O _water,VSMOW_ = −0.21 T(°C) +4.05

Marine: δ ^18^O_otolith,__VPDB_ - δ ^18^O _water,VSMOW_ = −0.21 T(°C) +3.72

The temperature experienced by a fish during otolith growth can therefore be estimated from otolith δ^18^O values, providing an estimate of the isotopic composition of oxygen in the ambient water is available (Trueman *et al.,* 2012). In marine waters, δ ^18^O values vary primarily according to mixing between fresh and sea water or with the degree of evaporation. Salinity is therefore a strong predictor for seawater δ ^18^O values, and databases of the relationship between surface water δ ^18^O values and salinity, and gridded datasets of extrapolated surface water δ ^18^O values are available (e.g. LeGrande and Schmidt, 2006). δ^18^Ο values in fresh and brackish waters may be variable at smaller temporal and spatial scales, influenced by local factors including rainfall, snowmelt and local evaporation. δ^18^Ο values in freshwater are typically lower than marine waters and increasingly so with distance from the ocean, as well as latitude and altitude due to temperature dependent Rayleigh distillation effects during evaporation and condensation (Yang *et al.,* 2019). Freshwaters are typically more accessible than marine waters for direct sampling, however, and global gridded spatial models of δ^18^Ο values in rainfall (e.g. Terzer *et al.,* 2013) also provide good initial estimates. Otolith based temperature estimates are therefore most reliable where the fish inhabits a water body that is isotopically constant and predictable during the time integrated by otolith sampling. Fish inhabiting fluctuating water bodies or moving across isotopic (salinity) gradients during the timeframe of otolith growth will present challenges for isotope-based temperature estimation.

**Figure 2:**
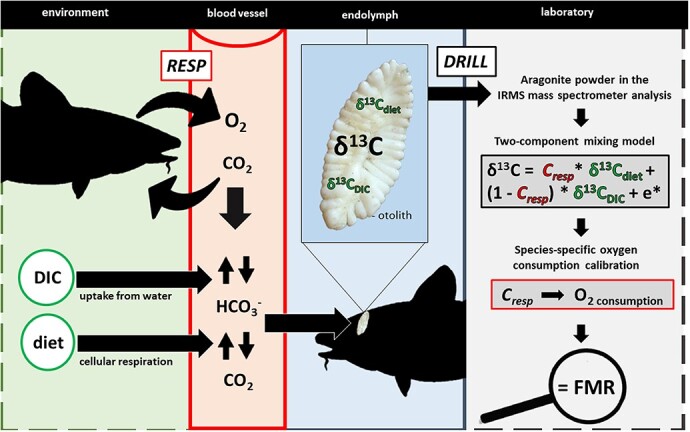
The stable otolith-isotope method is dependent on biochemical processes, in which respiration alters the carbon concentration of two isotopically distinct sources of carbon in the blood (δ^13^C_DIC_, δ^13^C_diet_ values), which then undergo fractionation and are deposited into the otolith as an interpretable stable isotope signature (Solomon *et al.,* 2006; Chung *et al.,* 2019a). The stable isotope signatures from DIC and diet are isotopically distinct, and the proportion of carbon derived from metabolic sources (C_resp_) can be isolated using a two-component mixing model. C_resp_ can then be converted to O_2_ consumption using a species-specific statistical calibration equation (Chung *et al.,* 2019b; Martino *et al.,* 2020), and converted to field metabolic rate (FMR). The abbreviation RESP stands for respiration (O_2_ consumption, CO_2_ production) and the e* in the two-component mixing model stands for the fractionation coefficient. The panels surrounded by solid lines represent processes that occur within the organism, while the dashed lines represent those outside the organism. (Atlantic cod silhouette: Cerren Richards).

The carbon precipitated as aragonite in the fishs' otolith is derived from two isotopically distinct sources: 1) dissolved inorganic carbon (DIC) from the surrounding water incorporated through the gill and/or the gut (δ^13^C_DIC_ value); and 2) metabolically derived carbon from the cellular respiration of food (δ^13^C_diet_ value) (Fig. 2: Kalish, 1991; McConnaughey *et al.,* 1997; Chung *et al.,* 2019a).

### 3.2. The Otolith/Field Metabolic Rate Link

Oxidation of food generates CO_2_, and in teleost fishes, approximately 90% of respiratory CO_2_ is carried in the plasma as HCO_3_. Consequently, despite high total carbon concentrations, the partial pressure of CO_2_ in blood of aquatic non-airbreathers is relatively low. Stable isotope data showing near equilibrium between ambient water and biomineral carbon in aquatic organisms imply that weak CO_2_ pressure gradients between blood and ambient seawater coupled with high ventilation rates (due to low ambient oxygen concentrations in seawater) result in diffusive ingress of ambient CO_2_ from the external seawater into blood at the respiratory surface (McConnaughey *et al.,* 1997; Solomon *et al.,* 2006; Tohse and Mugiya, 2008). Consequently, carbon from both metabolic and external sources is mixed in blood plasma. To maintain steady state, excretion of CO_2_ must match the combined influx of CO_2_ from respiration and ingress at the respiratory surface. As the rate of production of respiratory carbon increases, the partial pressure of CO_2_ in the blood increases and the rate of ingress of CO_2_ at the respiratory surface decreases. The proportion of metabolically derived carbon in blood (and ultimately otolith aragonite) then increases with increasing respiratory CO_2_ generation and provides a proxy measure for an organism's metabolic rate (Fig. 2, Kalish, 1991; McConnaughey *et al.,* 1997; Solomon *et al.,* 2006, Trueman *et al.,* 2013, 2016).

In marine fishes, the stable isotope composition of carbon in diet (δ^13^C value) is typically 15‰ to 30‰ lower than that of dissolved inorganic carbon (δ^13^C_DIC_ ranging between ~ −1‰ and 1‰ while diet carbon typically ranges between c. −15‰ and −30‰, respectively (Sherwood and Rose, 2005; Tagliabue and Bopp, 2008). If the δ^13^C_DIC_ and δ^13^C_diet_ values are known or can be inferred, the proportion of carbon in the otolith derived from metabolic sources (C_resp_) can be distinguished using a two-component mixing model (Chung *et al.,* 2019a, 2019b; Martino *et al.,* 2020):


(1) \begin{equation*} {\delta}^{13}{C}_{oto}={C}_{resp}\times{d}^{13}{C}_{diet}+\left(1-{C}_{resp}\right)\times{d}^{13}{C}_{DIC}+{e} \end{equation*}

Where *e* represents the total isotopic fractionation in* C *between bicarbonate in blood and *C* in aragonite. The value of e is unknown, some researchers apply a value of ~ 2.7‰ based on equilibrium fractionation of *C* during aragonite precipitation (Martino *et al.,* 2020, Smoliński *et al., *2021), whereas other studies apply a value of 0‰ reflecting mean overall fractionation between *C* sources and otolith aragonite observed in experimental studies (Solomon *et al.,* 2006; Chung *et al.,* 2019b; Alewijnse *et al.,* 2021). Critically comparative compilations of *C_resp_* values must employ a common *e* term.

High values of *C_resp_* indicate a high proportion of metabolic carbon in the otolith and consequently a high field metabolic rate. In the past, field studies have linked δ^13^C_oto_ values to alternative proxies for metabolic rate metabolism by examining swimming capacity in marine fish (Sherwood and Rose, 2003), or by examining the intra-otolith variations in δ^13^C_oto_ value in the temperature-driven metabolic cycles of archived freshwater drums (*Aplodinotus grunniens*; Wurster and Patterson, 2003).

Progress over the last 30 years has advanced the application of otolith δ^13^C values as a metabolic proxy, demonstrating robust and consistent relationships between the proportion of metabolic carbon in the otolith (*C_resp_* values) and fish body size, temperature and activity (Kalish, 1991; Wurster and Patterson, 2003; Sherwood and Rose, 2005; Solomon* et al.*, 2006; Trueman *et al.,* 2013; Sturrock et al., 2015; Chung *et al.,* 2019a etc.). Otolith *C_resp_* values have recently been used to demonstrate differences in FMR among sympatric ecotypes of cod (Chung *et al.,* 2021), to infer relative FMR among myctophid species (Alewijnse *et al.,* 2021), and infer historic variations in FMR in northern cod populations (Smoliński *et al.,* 2021).

Studies described above demonstrate that δ^13^C_oto_ and *C_resp_* values provide an effective proxy for relative FMR among and within individuals. The full potential of the otolith *C_resp_* method can only be realized if (a) *C_resp_* values can be calibrated against alternative measures of metabolic rate and (b) the potential for taxon-specific differences in the relationship between δ^13^C_oto_ values and metabolic rate is fully explored. Recent validation studies have strengthened and solidified the relationship between oxygen consumption, otolith carbon stable isotope signatures and metabolic metrics by using intermittent-flow respirometry to directly relate individual-level measurement of oxygen consumption to a δ^13^C_oto_ value in laboratory reared Atlantic cod (*Gadus morhu*a; Chung *et al.,* 2019b) and Australasian snapper (*Chrysophrys auratus*; Martino *et al.,* 2020) and presented in the form of exponential decay models:


(2) \begin{equation*} {C}_{resp}=C\ast \left(1-{e}^{-k\ast \left({FMR}_{oto}\right)}\right) \end{equation*}

Where the *C* and *k* terms are model fitted values, and FMR_oto_ is the oxygen consumptiopn rate in mgO_2_ Kg^−1^ h^−1^. The exponential decay model was chosen as in experimental manipulations where metabolic rate was modified by increasing temperature, increases in SMR at high temperatures were not matched by increase in observed *C_resp_* values (Chung *et al.,* 2019b). However, the non linear component is driven by a small number of individuals observed at high temperatures in the Chung et al experiment, and an alternative linear model (Eq 3) fits most observations well and is a better fit to group medians within temperature treatments (Chung *et al.,* 2019b). Furthermore, compilations of observational data from a wide range of fishes (Alewijnse *et al.*, 2021) show that relationships between *C_resp_* values and expected FMR are well approximated by a common linear calibration model. It is likely, therefore, that the decaying exponential model may in part reflect inclusion of additional metabolic components to SMR at high induced temperatures, coupled with variations in short term metabolic rates at the level of indidvidual expressed in respirometry but not in the longer-term integrated measure of FMR expressed in C_resp_ values (Martino *et al.,* 2020). The linear form of the calibration model presented in Chung *et al.* (2019b) avoids these potential artefacts and represents the most robust generalised calibration between C_resp_ and oxygen consumption rates.


(3) \begin{equation*} FMR_{oto}=\frac{{C}_{resp}-0.041}{0.000971} \end{equation*}

Additional experimental studies are crucial for validating calibration(s) between *C_resp_* values and other proxy (or direct) measures of FMR, identifying the extent of taxon-specific variation in the relationship between FMR and *C_resp_* values and constructing physiologically-based models to predictively describe the relationship between metabolic rate, gas exchange, and otolith δ^13^C values.

Isotopic analysis of otolith aragonite typically proceeds via evolution of CO_2_ gas following acid dissolution. Each analysis simultaneously provides δ^18^O and δ ^13^C values for the same otolith sample. The ability to examine the otolith δ^18^O value alongside the δ^13^C value therefore allows us to infer both the temperature experienced by the individual fish, and the FMR expressed at this temperature, averaged over the same period. The incremental structure of the otolith provides additional information relating to growth achieved under each observed combination of temperature and FMR. Because the otolith is an inert acellular structure, it does not undergo metabolic reworking or turnover (Thorrold *et al.,* 1997; Campana, 1999; Solomon *et al.,* 2006). Collecting the otolith at any point during a fish's lifetime provides an uninterrupted record of somatic growth linked to experienced water temperature and metabolic rate (i.e. feeding level). Combined information on realized growth in response to experienced temperature and expended energy may be particularly valuable for age estimations (Gauldie, 1996), life-history trait reconstruction (Gao *et al.,* 2010; Javor and Dorval, 2014), and determining the effects of anthropogenically altered environments on fish life histories (Schloesser *et al.,* 2009; Fraile *et al.,* 2016) or studying fishes that live in remote environments, such as the deep sea (Shephard *et al.,* 2007; Trueman *et al.,* 2013).

Otoliths are inert mineral structures, and the isotopic composition of carbon and oxygen in otolith aragonite is relatively robust to storage conditions, burial (Disspain *et al.,* 2016) and, in some cases, fossilsation (Patterson, 1999). Otoliths therefore also offer a method to explore metabolic ecology in historic, archaeological and palaeontological materials (Schutkowski *et al.,* 1999; Wurster and Patterson, 2003; Disspain *et al.,* 2016), providing long term perspective on evolutionary ecophysiology and fish responses to human -induced ecological and environmental disturbance.

## 4. Limitations, Considerations and Potential Solutions

The use of the otolith as a biogeochemical indicator of lifetime metabolic histories in wild fish provides a comprehensive tool for ecologists and physiologists alike, however, inherent limitations and logistical constraints call for further research effort to confidently use this method between and within species. Variation in diets, lifestyles and locations/migratory patterns may complicate the precision of estimates of δ^13^C values for diet and DIC, which could yield inaccurate estimates of individual FMR. Moreover, applying this method to wild fishes in a macroecological framework might benefit from species-specific laboratory validations. Here, we provide a brief summary of key limitations and developing solutions in relation to the major steps associated with the δ^13^C_oto_ method: δ^13^C_DIC_ and δ^13^C_diet_ value variation, fractionation, *C_resp_* determination and oxygen consumption estimation, while a comprehensive review is available in Chung *et al.* (2019a).

### 4.1. **C**_**resp**_ **Determination**

The proportion of metabolically sourced carbon in the otolith (*C_resp_*) of an individual is dependent on the accuracy of estimates of both δ^13^C_DIC_ and δ^13^C_diet_ values in the two-component mixing model (Fig. 2). Past studies on a variety of fish species (summarized in Chung *et al.,* 2019a) have determined that *C_resp_* values estimated from two-component mixing models generally fall in the range of 0–0.5. It is currently uncertain whether *C_resp_* values that fall outside of this range yield inaccurate *C_resp_* estimations (Wurster and Patterson, 2003; Hanson *et al.,* 2013), or whether these are consistent with species-specific metabolic lifestyles, such as the high metabolism experienced by tuna.

#### 4.1.1. **δ** ^**13**^ **C**_**DIC**_ **Value Variation**

The proportion of respiratory carbon in otoliths is typically less than 30%, therefore, estimates of *C_resp_* values are more sensitive to uncertainty in δ^13^C_DIC_ values than to δ^13^C_diet_ values, which is helpful in marine settings where δ^13^C_DIC_ values are better constrained. Variation in water δ^13^C_DIC_ values occurs based on the environmental source(s) and isotopic compositions of dissolved organic carbon, as well as the extent and of type of photosynthesis involved and the size of the water body (in essence the degree of isotopic buffering). In open marine systems, DIC levels remain relatively uniform between c. −0.5‰ and 1.5‰ due to the carbonate buffer system. However, temperature-driven differences in the solubility of CO_2_ in seawater and variations in the rate of removal of CO_2_ through photosynthetic fixation impose systematic variation on oceanic δ^13^C_DIC_ values. Databases and models (Tagliabue and Bopp, 2008; Schmittner *et al.,* 2013) describing oceanic δ^13^C_DIC_ values provide reasonable estimates (and uncertainties) suitable for otolith *C_resp_* mixing models. Non-oceanic, coastal, estuarine and freshwater systems are typically more seasonally and spatially variable, however, resulting in highly variable water DIC concentration and δ^13^C_DIC_ values. Larger lakes may exhibit relatively seasonally stable δ^13^C_DIC_ values, assisting the application of otolith carbon isotopes to infer metabolism (Solomon *et al.,* 2006; Weidel *et al.,* 2007; Gerdeaux and Dufour, 2015).

**Figure 3:**
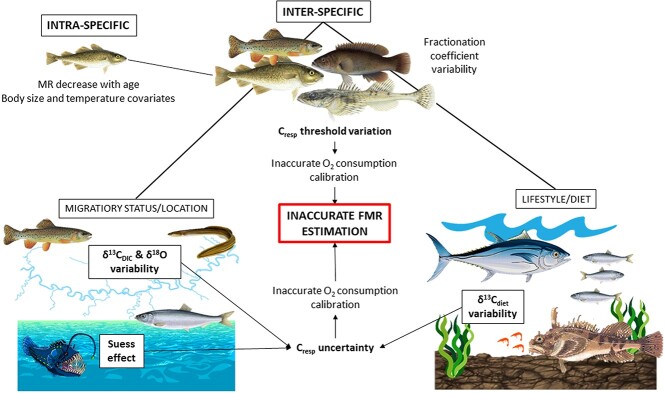
A conceptual illustration of the limitations to consider when conducting studies aimed at examining differences in FMR through an intra-specific and inter-specific lens. The inter-specific category is further divided into migratory status and location (migrating, freshwater, marine, deep-sea), as well as lifestyle and diet in order to highlight specific processes that might affect the accuracy of FMR estimation.

While δ ^13^C_DIC_ values are relatively uniform in oceanic environments (especially the deep-sea; Tagliabue and Bopp, 2008; Schmittner *et al.,* 2013; Becker *et al.,* 2016), temporal effects also cause variation in the δ^13^C_DIC_ values. The Suess effect (Fig. 3) is important to consider when making use of otoliths from historic archives and comparing these over long time periods and is reflected by the reduction of the δ^13^C value of atmospheric carbon resulting from the burning of fossil fuels with relatively low δ^13^C values (Keeling, 1979; Eide *et al.,* 2017; Chung *et al.,* 2019a). There is a mismatch between the atmospheric and biological effects of the Suess effect over time, in which amplified phytoplankton growth rates increase the biological fractionation of carbon, reducing the δ^13^C values of organic carbon more rapidly than those of DIC. While there are good records of the extent of the atmospheric and biological effects of the Suess effect across ocean basins (e.g. Körtzinger *et al.,* 2003; Tagliabue and Bopp, 2008; Young *et al.,* 2013; de la Vega *et al.,* 2019), this discrepancy should be addressed when conducting analyses involving FMR comparisons across large timeframes using historical otolith records.

#### 4.1.2. **δ** ^**13**^ **C**_**diet**_ **Value and Fractionation Variation**

The isotopic composition of diet carbon sources can be estimated from stable isotope analyses of muscle tissue of the fish under study, or from compilations of isotopic data for prey sources. As diet contributes proportionally less carbon to the otolith than ambient water, uncertainty in δ^13^C diet values will contribute less to overall uncertainty, particularly in less active fishes where the contribution of dietary carbon to the otolith is minimised (Fig. 3; Chung *et al.,* 2019a). *C_resp_* values are calculated from a two-component mixing model (e.g. equation 1). Uncertainties in all terms can be quantified and incorporated in estimates of *C_resp_* values. Bayesian mixing models such as mixSIAR (Stock *et al., *2018) can be used to estimate credible intervals on *C_resp_* values at an individual level (Alewijnse *et al.*, 2021), or Monte Carlo resampling approaches can be used to estimate a common uncertainty across a population or study (Stock *et al.,* 2018; Chung *et al.,* 2019a).

#### 4.1.3. **C**_**resp**_ **and Oxygen Consumption**

As discussed above, the relationship between *C_resp_* values and oxygen consumption rates is uncertain, and additional experimental and theoretical work is needed to constrain calibrations and to evaluate the extent to which a common calibration can be applied across taxa. In the absence of experimental data, the linear form calibration presented in (Chung *et al.,* 2019b) is more applicable across taxa than the exponential decay form. However, *C_resp_* values serve as a valuable proxy for metabolic rate independent of conversion to equivalent oxygen consumption rates.

### 4.2. Intra-specific Variation

While uncertainty can arise during the conversion of *C_resp_* to alternative proxies for FMR, variation in lifestyles, diet, or locations, there are also considerations to keep in mind when conducting FMR comparisons between individuals or populations of the same species (Fig. 3). Body size and temperature are important covariates to influencing all metrics of metabolic rate and should be similarly considered for the FMR proxy. Body size has a major influence on metabolic rate, as energy demands increase allometrically with biomass (Glazier and Glazier, 2005; Killen *et al.,* 2010). While the otolith provides an opportunity to measure the long-term time integrated metabolic rate in wild fish, limitations are present when making intra-specific comparisons across life stages. For example, the metabolic rate of fish is influenced by a mass-specific decrease with age, which is reflected in the otolith as an increase in δ^13^C_oto_ values across its lifetime (Fidhiany and Winckler, 1998; Rosenfeld *et al.,* 2015; Peck and Moyano, 2016; Trueman *et al.,* 2016; Chung *et al.,* 2019a). Sampling should therefore be focused on individuals of the same size and temperature exposure, or alternatively, a scaling relationship should be determined to correct for differences in temperature and size effects (Chung *et al.,* 2019a).

Water temperature is another factor that can influence FMR estimates and changes rapidly in coastal systems with marked variation across seasons. The ability to analyse the otolith δ^18^O value alongside the δ^13^C value, however, allows for the reconstruction of time-specific experienced temperature in wild fishes (Kalish, 1991; Kitagawa *et al.,* 2013). Otolith isotope-derived FMR values can therefore be standardized in accordance with individual-specific experienced temperature and season, allowing for confident estimations of temperature-influenced processes, such as food conversion efficiency, and growth potential at coarser time scales.

## 5. Analytical Flexibility: Determining the Resolution and Precision of Field Metabolic Rate Data

Pairing the recording capacity of the otolith's structure with differing otolith processing techniques would allow researchers to use the otolith to target trends at varying time scales and resolutions. The precision applied during aragonite powder extraction within or across time-integrated mineral bands determines the temporal scale covered by the isotopic environmental tracers (days-lifetime). While other techniques of micro-sampling are potentially available (SIMS, ion microprobe analysis, laser ablation techniques), these are generally not suitable for biomineral δ^13^C analysis as measurements are confounded by the presence of the organic matrix which is also ablated during probe-based sampling.

**Figure 4:**
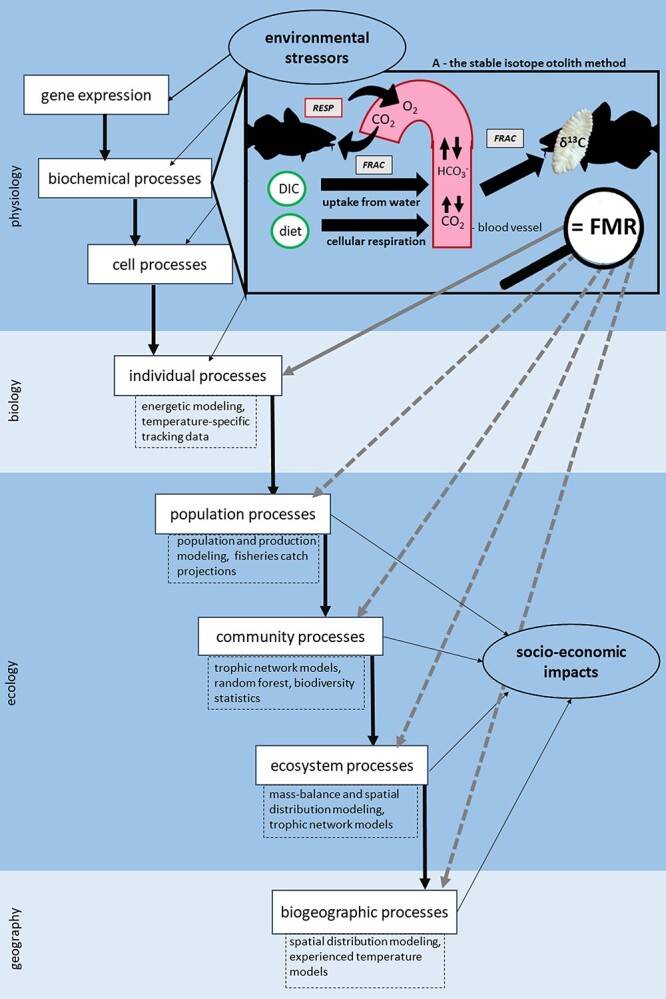
A conceptual illustration identifying where the otolith-isotope method fits into the hierarchical levels of biological complexity (A. biochemical processes) and provision of FMR information at various scales, which can in turn be incorporated into existing resource management models. Processes are identified as interlinked boxes overlaid on a background (light and darker blue) of increasing biogeographic scale moving down the figure. Impacts of environmental stressors on, and socio-economic outputs from, these processes are indicated by thin black arrows. The thick grey arrows depict the ability to utilize otolith-derived FMR data to directly (solid) and indirectly (dashed) predict processes at multiple levels of organisation. This figure has been adapted from Le Quesne and Pinnegar (2011) and Metcalfe *et al.* (2016). (Atlantic cod silhouette: Cerren Richards).

The appropriate temporal resolution (i.e. choice of otolith growth over which to integrate a sample) depends on the research question under investigation. Hand drilling otolith outer surfaces provides the highest temporal resolution as the full surface area of the otolith is available for sampling. Micromilling sectioned otoliths allows multiple sampling over ontogeny for a single individual (Weidel *et al.,* 2007; Chung *et al.,* 2019b). Serial sampling or temporally targeted sampling provides an opportunity to capture retrospective estimates of metabolic costs associated with long-term natural behaviours (seasonal movements, reproduction, established trophic interactions) over life history periods of interest (ontogeny), or site-specific environmentally-significant time periods (El Niño, overwintering periods, chronic eutrophication, long-scale habitat alteration projects, local anthropogenic/agricultural runoff). However, sampling from otolith sections generally comes at a cost of lower temporal resolution and with less precise estimates of body size compared to sampling otolith edges. The ability to examine the FMR proxy over a long timescale can be beneficial in minimizing the impact of extreme or abnormal behaviours on net *C_resp_*, allowing for the analysis of averaged, yet robust, trends at several levels of biological organization (Fig. 4). While obtaining a weighted average *C_resp_* value over a larger time period can be ecologically relevant, such measures warrant additional caution, as temporal covariance between temperature and FMR could be mismatched.

Otolith processing techniques that increase the temporal resolution of the FMR proxy present an opportunity to examine daily periodicity of metabolic rate and experienced temperature to target the acute physiological effects of short-term environmental (weather, plumes, upwelling, environmental/anthropogenic runoff, acute eutrophication) or behavioural (transition between life history stages, short-term migration, prey selection, spawning, habitat choice) dynamics (Weidel *et al.,* 2007). Targeting *C_resp_* over short-time periods at a high resolution provides an alternative to long-term FMR-proxies, which might not have the appropriate high frequency environmental sampling data to support studies aimed at analysing physiological responses to climate (Weidel *et al.,* 2007). The ability to examine acute physiological trends at high resolutions without the need for large-scale environmental data integration is also valuable for examining archived otoliths, allowing for the reconstruction of thermal histories and energetic time-series as useful indicators of past climates.

The temporal resolution is also important in otolith-isotope based FMR estimates and relies on the amount of aragonite powder that can be removed and collected for analysis (isotope ratio mass spectrometry (IRMS) typically requiring c. 5 to 50 μg powder) and will translate to different timescales of otolith growth based on otolith size and fish growth rate. While otolith size has historically limited high-resolution isotope profiles over narrow timescales, recent advances in otolith processing techniques have allowed for increased precision during both the aragonite extraction and isotope analysis phases. The smaller amounts of aragonite material obtained by milling along the otolith growth axis has previously limited the maximal resolution of metabolic information by both the growth rate and the size of the otolith, and therefore, high-frequency time-series were constrained to fish with notably large otoliths (Wurster and Patterson, 2003; Wurster *et al.,* 2005; Chung *et al.,* 2019b). Sampling the outer surface of otoliths (as opposed to sectioned otoliths) maximises the surface area available for sampling and reduces uncertainty in estimates of water δ ^13^C_DIC_ and δ ^18^O values as the smaller timeframe sampled relates more directly to the location at which the fish was caught.

## 6. Incorporating Otolith-Isotope Inferred Energetics into Conservation Strategies

Most studies exploring the otolith FMR method to date have focused on demonstrating the link between otolith δ^13^C values and metabolism (Kalish, 1991), calibrating against alternative respiration proxies (Chung *et al.,* 2019b; Martino *et al.,* 2020), and testing the sensitivity of the approach relative to natural ontogenetic and ecophysiological variations (Jamieson *et al.,* 2004; Trueman *et al.,* 2013; Niloshini Sinnatamby *et al.,* 2015). Direct application of otolith derived FMR estimates to inform conservation strategies remains largely a potential. One clear example is given by Trueman *et al.* (2023) working on Atlantic bluefin tuna. This project used otolith-inferred C_resp_ and δ ^18^O values to build thermal performance curves for FMR for year 0 Atlantic bluefin tuna from the western Atlantic (Gulf of Mexico, SW Seaboard) and Mediterranean regions. Atlantic bluefin tuna showed a clear thermal optimum for FMR at 28°C, and Trueman *et al.* (2023) inferred that limited availability of water < 28°C explains relatively weak recovery of western compared to eastern tuna populations. Furthermore, drawing on climate model projections of warming in the Mediterranean, Trueman *et al.* (2023) proposed timeframes for thermal limitation of juvenile tuna in the Mediterranean and their consequent earlier movement into the European Atlantic with implications for interaction with existing pelagic fisheries.

The otolith-isotope method forms a link between the individual's physiological response and its greater environment by suggesting impacts at higher levels of biological organization, in this case, being changes in ecotype-specific competitive interactions within populations (Fig. 4). For instance, Chung *et al.* (2021) quantified differences in field metabolic rate and its thermal sensitivity in sympatric juvenile cod ecotypes. In doing so, they identify differential sensitivities to warming in genetically distinct populations, highlighting that conservation strategies should account for potential differences in ecophysiological traits at population level. Otolith-derived FMR estimation is an attractive tool as it avoids population specific acclimation effects that can complicate laboratory-based assessments of thermal sensitivity.

The latest version of the widely accepted Conservation Standards assembled by the Conservation Measures Partnership has stressed the importance of assessing individual physiological response to warming (Seebacher *et al.,* 2023). The thermal sensitivity of FMR and the mean FMR of individuals contributes to this call by providing an important foundation for energetic modelling at the population level (fish growth and viability— population/production modeling, fisheries projection models), as well as the community and ecosystem level (distribution/habitat use—trophic network models). The application of this method has also recently been expanded to historical otolith collections (Smoliński *et al.,* 2021), examining differences in FMR between Atlantic cod (*Gadus morhua*) populations from Iceland and the Barents Sea over a nearly a century (1914–2013). Studies are also expanding the otolith FMR method to other species, for example estimating relative field metabolic rates in myctophid fishes to their role in ocean carbon export (Alewijnse *et al.,* 2021). This first *in situ* use of the otolith-isotope method provides a clear example of how field measurements provide an analytical framework to inform conservation and management strategies, specifically in assessing the extent to which climate change may influence the viability of conservation targets (Seebacher *et al.,* 2023).

Monitoring individual response to complex environmental stressors is important in determining the underlying mechanisms that drive the trend toward either vulnerability or resilience in the face of climate and anthropogenic change. Resilience can be further divided into two pathways—that of resistance to, or recovery from, destabilizing anthropogenic and climatic stressors. Organisms with resilient metabolic genotypes are selected for in a population, providing a basis upon which natural selection can act (Gunderson and Stillman, 2015; Martínez *et al.,* 2015). The metabolic responses of individuals to environmental stressors can highlight important interindividual variability in physiological traits within and across populations, in which higher levels of physiological diversity is synonymous with higher resilience (Spicer and Gaston, 2009). Physiological diversity represents the variability in physiological traits (metabolic rate, hemoglobin-oxygen affinity, thermal tolerance, osmoregulatory ability, resting membrane potential) among organisms in a population or community (Spicer and Gaston, 2009). This physiological diversity does not only stem from responses to direct environmental stressors, but also from unique genetic and developmental factors (Spicer and Gaston, 2009). Physiological performance of individual or populations measured through the otolith-isotope method (oxygen consumption, FMR) can highlight gaps or successes when making conservation targets in regards to individual performance and population persistence (Seebacher *et al.,* 2023). Understanding how the underlying physiological responses of individuals are situated within higher levels of biological organization is crucial to create realistic predictions of ecosystem vulnerability or resilience. Furthermore, since climate threats are unpredictable and do not necessarily follow latitudinal gradients, the determination of physiological vulnerability at regional and local scales might reveal climate risks that could otherwise go undetected (e.g. Burrows *et al.,* 2011).

Otolith-isotope inferred FMR-proxies can provide insight into the energy requirements, physiological responses, behavioural adaptations and trophic interactions of free-ranging fishes exposed to environmental change and anthropogenic disturbance (Nagy *et al., *1999; Chung *et al.,* 2021; Martino *et al.,* 2020). It is difficult to use field metabolic rate alone as an indicator for anthropogenic and climatic physiological stress, however, combining stress indicators at multiple levels of biological organization can start to paint a holistic picture of *in situ* impacts. Combining individual-level examinations of metabolic rate fluctuations with population-level analyses of reproductive output and physiological diversity can create context for the allocation of energy associated with the field metabolic rate value.

**Figure 5:**
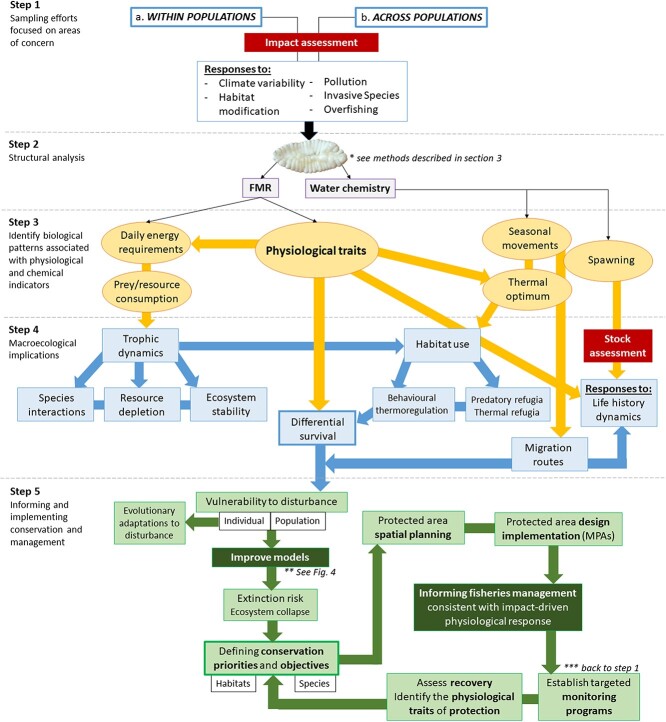
Phases for integrating the otolith-isotope method into conservation strategies. These phases include: (Step 1) determining whether comparisons will be made within populations or across populations, subsequently dictating the sampling effort and size associated with fieldwork, as well as the targeting of individuals in areas under anthropogenic or climatic pressure; (Step 2) conducting the isotopic analysis of the otolith; (Step 3) identifying biological patterns associated with FMR and water chemistry and employing any necessary calibrations; (Step 4) creating links between biological and macroecological processes; (Step 5) implementing vulnerability, structure, and system response into energetic models to inform conservation objectives and lead protected area design and sustainable fisheries initiatives.

Figure 5 connects the many steps associated with the otolith-isotope method and its ability to address research questions from various fields (physiology, toxicology, animal behaviour, evolution, food web ecology) to inform conservation strategies. Because of the inherent limitations associated with interspecific studies, Step 1 will restrict the initial sampling effort to make comparisons between individuals and populations of the same species in areas influenced by climate variability or anthropogenic change. The FMR (δ^13^C) and water chemistry (δ^18^O) values obtained from the otolith record serve as indicators for biological processes, such as daily energy requirements and seasonal movement, respectively, which in turn can be linked to larger-scale ecological dynamics (Fig. 5: Steps 2 and 3). The intra-specific physiological stressors associated with fluctuating environments can influence species interactions, trophic dynamics, habitat use, and life history stages, providing crucial *in situ* information (Step 4) for the development of effective conservation strategies (Fig. 5: Step 5).

The unique precision of the otolith as a spatiotemporal tool linking FMR to experienced temperature and location provides four key interlinked opportunities to inform and advance conservation and management strategies:

**1. Physiological response to regional climate pressures.** The otolith-isotope method can be applied to quantify the impact of local (e.g. agricultural/industrial runoff, eutrophication) and wide-scale (acidification, El Niño events, temperature, heat waves) climate stressors on the FMR of free-ranging fishes. Moreover, the otolith's ability to elucidate FMR data with a spatiotemporal lens allows for a three-dimensional assessment of climate impacts—i.e. examining the influence of environmental fluctuations on individual metabolism across heterogeneous environments and seasonal, inter-annual and decadal timescales. Understanding how acute and chronic climate stressors alter the physiological diversity of natural populations over space and time can provide insight on the metabolic and behavioural strategies adopted by individuals and populations in the face of climate variability with the potential for behavioural thermoregulation, niche variation, range expansion and alterations in migratory routes, which all have cascading impacts on wider ecosystem processes. The capacity to track how the critical physiological phenotypes of individuals differ within and between populations can inform conservation objectives on the vulnerability or the inherent resilient responsivity of populations under different climate stressors. *In situ* data on physiological costs therefore allow for informed protected area spatial planning consistent with the vulnerability of populations in the face of climate change.

**2. Physiological response to local anthropogenic stressors.** Additional drivers of physiological stress can be identified by using the otolith-isotope method to target populations impacted by anthropogenic pressures, such as habitat modification (shoreline development, dam and wave energy conductor construction), species exploitation (commercial or recreational fishing, changes in predator abundance), or pollution exposure (pesticides, metals, pharmaceuticals, light, sound). An opportunity arises to use the otolith-isotope method as a tool to produce warning signals of harmful and irreversible anthropogenic pressure on individuals under climate stress. This method allows us to identify whether local anthropogenic conditions amplify or dampen the effects of climatic stressors. For example, questions regarding the influence of urbanised versus natural habitats, or varying fishing intensity, on the thermal optimum for FMR or the metabolic phenotypic diversity of a population can be explored. For instance, the otolith records the energetic costs of free ranging fish over large temporal scales and thus provides a unique opportunity to examine how sudden alterations in human activity affects the energetic budgets of fish populations. How a system responds to direct and indirect anthropogenic stressors can provide further insight into the behavioural and physiological adaptations involved in driving resilience (resistance or recovery), informing stakeholders and policy makers on any necessary alterations to anthropogenic pressures to ensure the health of overall ecosystems and the sustainable productivity of fish stocks.

**3. Improve energetic models.** Examining individual variation in FMR provides insight on the full energetic budget of a free-ranging fish exposed to real-time climate variability and anthropogenic pressure, with the ability of examining the potential physiological costs associated with extreme temperatures (critical thermal limits) and resource depletion (daily energy requirements). This valuable *in situ* data increases the predictive power of modeling efforts at various levels of biological organization, including energetic models, population and productivity models, fisheries-catch projections, spatial distribution models and trophic network models (Fig. 4). By integrating real-time *in situ* data into these models, they will provide better estimates of the long-term growth and productivity of populations under current and projected climate scenarios, with the ability of including the additional physiological costs associated with anthropogenic activity to assess real-time ecosystem stability.

**4. Assessing conservation efforts.** The otolith-isotope method can inform and implement marine protected areas and maximum sustainable yield (MSY) estimates. This method can also be used to assess differences in physiological phenotypes between protected and unprotected populations. Incorporating the otolith-isotope method into long-term monitoring programs aimed at quantifying the physiological performance of protected versus unprotected populations can provide valuable information on the effectiveness of conservation initiatives and can help weigh the direct costs and benefits of implementation.

A prime **example** of the four key opportunities associated with the otolith-isotope method described above is its application to fishery science, with the aim of providing well-informed projections that ensure consistency between the real-time metabolic capacity of fish populations, fishing efforts, stock management, and conservation objectives. (1) Real-time *in situ* data on the metabolic vulnerability of fish populations to climate events provide a unique opportunity to alleviate any additional pressures caused by (2) anthropogenic activities (e.g. fishing pressures) to ensure population persistence and ecosystem health. This means integrating *in situ* physiological costs into (3) energetic models and stock assessments to create maximum sustainable yield (MSY) estimates and fisheries catch projections that promote the maximum productivity of fish stocks under real-time environmental and anthropogenic pressures on fish populations. When combined with long-term fisheries catch projections informed by greenhouse gas emissions, the otolith-isotope method can provide valuable information on the physiological costs associated with acute climate events for regions of concern (Cheung *et al.*, 2011, Fig. 5). Incorporating *in situ* data into maximum sustainable yield estimates can inform conservation initiatives aimed at controling anthropogenic pressures on populations under climatic stress, while (4) subsequently encouraging management practices that ensure a sustainable equilibrium state between fisheries capture rates and fish population productivity. This could be achieved by integrating the otolith-isotope method into long-term monitoring programs. The energetic performance of populations under climate-informed fisheries practices can then be continually assessed to determine whether conservation and management practices actively contribute to reliable long-term global food security and ecosystem health.

## 7. Conclusion and Future Directions

The potential to examine the dynamic balance between maintenance and activity metabolism within the scope of FMR can highlight physiological and behavioural plasticity in the face of climate variability and anthropogenic stressors, with direct implications for species interactions and population dynamics (Chung *et al.,* 2021). Predicting the cascading effects of individual metabolism on behaviour is especially important in marine systems, with its relative lack of barriers to dispersal and greater colonization rates, as it provides insight into the metabolic drivers associated with species distribution, community compositional turnover and trophic interactions (Poloczanska *et al.,* 2013; Burrows *et al.,* 2019; Pinsky *et al.,* 2019).

The otolith-isotope method outlines an exciting path forward in the field of ecophysiology, allowing flexibility in answering *in situ* questions, such as the physiological costs of locomotion over specific migration routes, for example. This presents a novel opportunity for various branches of biology and physiology, as measuring the activity involved in trophic interactions, such as foraging and predation, is difficult within a laboratory setting, usually calling for the use of microcosm and mesocosm experiments, which are difficult to replicate, are costly, and can lead to oversimplification of ecosystem processes due to their narrow spatial and temporal scales (Coelho *et al.,* 2013). While mesocosms can demonstrate the metabolic balance of planktonic communities within imitated wetland and mangrove habitats (Oviatt *et al.,* 1986; Lv *et al.,* 2017), mesocosms have not been used to measure individual metabolism of vertebrates.

The measurement of FMR with the otolith-isotope method presents the first truly holistic view of the energetic costs associated with environmental change directly measured from free-ranging fish, providing insight on somatic growth, dietary breadth, experienced temperature, and metabolism. Future opportunities include applying the aragonite-based method to a wider range of taxa which incorporate metabolically derived carbon into their carbonate structures, such as mollusks (δ^13^C value: oxygen consumption) and foraminifera (δ^13^C value: symbiont photosynthetic activity), with interpretable deviations of ~ 5‰ between δ^13^C values and inorganic aragonite precipitation (Grossman and Ku, 1986; Prado Jr *et al.,* 2013). There is also potential to extend this method to extant and archived urchin species (Grossman and Ku, 1986; Spero and Williams, 1987; Prado Jr *et al.,* 2013) which incorporate metabolically derived carbon into their aragonite tests, previously been used for diet analysis (Prado Jr *et al.,* 2013). Similar assessments of other calcium carbonate based benthic consumers and photosynthesizers, such as crustaceans, gastropods and corals, could provide insight into field based metabolism. For instance, the relationship between δ^13^C signatures and respired CO_2_ to allow for species-specific calibrations to accurately depict metabolically derived carbon signatures, such as for photosynthetic organisms, e.g. foraminifera and coral (Swart, 1983; Høie *et al.,* 2003). Despite the inherent limitations associated with the otolith-isotope method, the increasing application of this method will mirror the development of new technologies, supporting various fields of ecophysiology and taxa, allowing scientists to refine their lens on the climatic and anthropogenic changes to aquatic environments and determine the best ways to move forward in mitigating them.

## Data Availability

No data were generated or analysed during this work.
